# Systematic Benchmarking of DNA Sequence Encoding Strategies for Predicting Regulatory Effects of Non-Coding SNPs

**DOI:** 10.3390/ijms27156657

**Published:** 2026-07-25

**Authors:** Hui Jin, Yihang Bao, Wenhao Li, Chengyi Yang, Weidi Wang, Wenxiang Cai, Zhe Liu, Guan Ning Lin

**Affiliations:** 1 School of Biomedical Engineering, Shanghai Jiao Tong University, Shanghai 200230, China; hui.jin@sjtu.edu.cn (H.J.); baoyihang@sjtu.edu.cn (Y.B.); liwenhao0504@sjtu.edu.cn (W.L.); sjtu_ycy@163.com (C.Y.); wwd@smhc.org.cn (W.W.); caiwenxiang@sjtu.edu.cn (W.C.); 2 Department of Computer Science and Engineering, East China University of Science and Technology, Shanghai 200237, China

**Keywords:** non-coding SNPs, encoding strategies, benchmarking, regulatory variants, DNA methylation, deep learning

## Abstract

Non-coding single nucleotide polymorphisms (SNPs) are key modulators of gene regulation and have been implicated in diverse complex traits and diseases. With the growing demand for accurate functional interpretation of non-coding variants, the choice of encoding strategies becomes critical in downstream predictive modeling. Despite recent advances, a systematic evaluation of encoding approaches tailored for non-coding SNPs remains lacking. To address this gap, we present a comprehensive benchmark that evaluates six representative encoding strategies, including categorical, semantic, and functional embeddings, across three quantitative trait loci (QTL)-related prediction tasks. The study encompasses nine machine learning and deep learning models and incorporates experimental controls and repeated trials to ensure robustness and reproducibility. We assess each strategy along multiple dimensions, such as interpretability, representation abundance, and computational efficiency. Rather than ranking individual methods, our analysis emphasizes the interaction between encoding strategies, model types, and preprocessing protocols, and highlights their collective influence on predictive performance. This work establishes a standardized framework for evaluating non-coding SNP representations and offers guidance for selecting and optimizing prediction pipelines in regulatory genomics.

## 1. Introduction

Non-coding single nucleotide polymorphisms (SNPs) are critical genomic variants that influence gene regulation and phenotypic diversity without altering protein sequence [1,2,3]. These variants play key roles in regulating transcription, splicing, and epigenetic modifications, making them central to understanding disease mechanisms and genetic architecture [4]. Despite their importance, decoding the functional impact of non-coding SNPs remains a challenging task due to their complex regulatory mechanisms and the subtle nature of their effects [5,6].

Advances in genomic datasets, such as expression quantitative trait loci (eQTL) [7] and methylation quantitative trait loci (meQTL) [8], have created unprecedented opportunities to investigate the regulatory roles of non-coding SNPs. eQTL datasets elucidate how SNPs influence gene expression, while meQTL datasets provide insights into their effects on DNA methylation, an essential epigenetic modification. These datasets enable researchers to explore multiple layers of SNP-mediated regulation. However, the complexity of these tasks, combined with the diverse properties of available datasets (e.g., sample size, tissue specificity), underscores the need for systematic evaluation to identify effective encoding strategies and modeling approaches.

Computational tools have been developed to predict these impacts by encoding non-coding SNPs into machine-readable formats [9,10,11,12], yet the relative strengths and limitations of various encoding strategies and modeling approaches remain underexplored. A crucial step in addressing this challenge is exploring how to encode non-coding SNPs into features that computational models can process effectively. However, there is currently no objective evaluation or comprehensive guideline for selecting or designing encoding strategies tailored to sequence-based downstream prediction tasks. This lack of clarity leaves researchers uncertain about which encoding strategies are most effective for specific tasks and under what conditions they perform optimally. Bridging this gap requires a systematic benchmark to compare different encoding strategies and assess their applicability in modeling non-coding SNPs.

Various encoding strategies have been developed to transform non-coding SNPs into machine-readable features, broadly categorized into categorical, functional, and semantic strategies. Categorical strategies, such as one-hot encoding, provide simple yet explicit representations of base-level information. Functional strategies, exemplified by models like Enformer [13], integrate genomic annotations to encode regulatory features. Semantic strategies, including pre-trained DNA language models like DNABert2 [14], Genomic Pre-trained Network (GPN) [15], HyenaDNA [16], and Nucleotide Transformer (NT) [17], leverage deep learning to capture context-dependent relationships within DNA sequences. Here, semantic refers to sequence embeddings learned from large-scale, self-supervised DNA language models that capture context-dependent and high-order relationships between nucleotides, analogous to semantic relationships in natural language. We categorize them together because they derive semantic meaning from nucleotide contexts rather than relying on handcrafted features or external annotations, distinguishing them from categorical or functional strategies. Each approach has distinct advantages and trade-offs, but their relative effectiveness in predicting downstream outcomes of non-coding SNPs, such as gene expression or DNA methylation changes, remains underexplored.

Recent reviews and benchmarks have systematically evaluated DNA language models and genomic foundation models across diverse biological tasks [18], highlighting both their potential and their task-dependent limitations. These studies suggest that model performance can vary substantially with task formulation, sequence context, downstream model design, and evaluation protocol.

In this work, we present a comprehensive benchmark study to evaluate six distinct DNA sequence-based encoding strategies, ranging from traditional categorical encodings to advanced pre-trained semantic models and functional annotation-based embeddings. These strategies are assessed across key downstream tasks, including eQTL and meQTL prediction, using both machine learning and deep learning models. Our study focuses specifically on how categorical, semantic, and functional encoding strategies perform for non-coding SNP representation in QTL-related prediction tasks. By systematically analyzing the impact of encoding strategies, model types, data sizes, and preprocessing strategies, we address critical questions about how to design prediction pipelines for non-coding SNPs. Our work emphasizes the need for clear guidelines in selecting encoding strategies and provides insights into optimizing performance across different genomic contexts.

## 2. Results

### 2.1. Overview of Benchmark Framework

**Figure 1:**
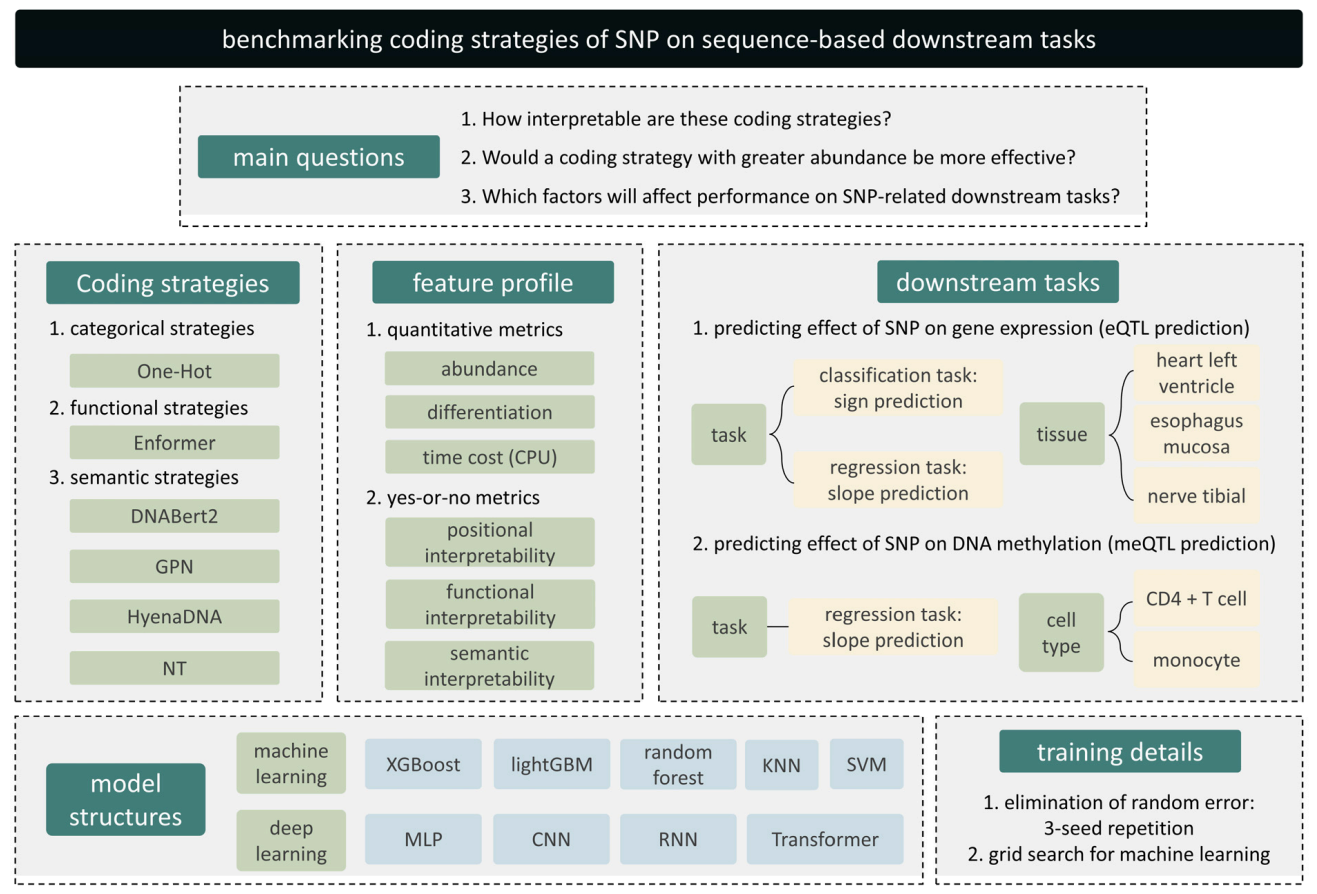
Framework for benchmarking encoding strategies of non-coding SNPs. This framework evaluates six distinct encoding strategies, categorized into categorical, functional, and semantic strategies. We designed six quantitative metrics to intuitively compare these strategies and assessed their performance across three quantitative trait loci (QTL)-related downstream tasks. During the evaluation, a controlled variable approach was employed, with key variables such as encoding strategies, machine learning/deep learning frameworks, tissue/cell types, data volume, and preprocessing strategies systematically observed.

Non-coding single nucleotide polymorphisms (SNPs) play a pivotal role in gene regulation and disease mechanisms. However, the optimal strategies for encoding non-coding SNPs in computational downstream tasks remain unclear. To address this, we designed a benchmarking framework (Figure 1) that seeks to answer three fundamental questions:

1. How interpretable are these encoding strategies?
2. Are encoding strategies with higher encoding abundance (representation dimensions per base) more effective?
3. What are the key factors influencing performance in downstream tasks?

Our approach categorized six widely used encoding strategies into three conceptual groups: categorical, functional, and semantic strategies. These include the traditional one-hot encoding method, the state-of-the-art sequence functional annotation representation model Enformer [13], and four pre-trained DNA language models: DNABert2 [14], Genomic Pre-trained Network (GPN) [15], HyenaDNA [16], and Nucleotide Transformer (NT) [17]. These strategies were selected to represent a spectrum of encoding philosophies, ranging from simple base representation to complex contextual embeddings.

To systematically compare these strategies, we defined six key metrics, or feature profiles, aimed at quantifying their strengths and limitations. These include the ability to differentiate SNP effects, computational cost, compatibility with pooling mechanisms for sequence aggregation, and three complementary dimensions of interpretability: contextual (sequence-level), functional (annotation-level), and semantic (biological insight-level). Together, these metrics provide a multidimensional perspective on how encoding strategies translate genomic information into predictive tasks.

We applied this framework to three quantitative trait loci (QTL) [19,20]-related downstream tasks, investigating how non-coding SNPs influence gene expression (eQTL prediction, classification and regression tasks) and DNA methylation levels (meQTL prediction, regression task). These tasks spanned diverse tissue and cell types, incorporating both classification and regression objectives to ensure a holistic evaluation. Importantly, we considered not only the encoding strategies themselves but also the interactions between these strategies and model architectures. To this end, we tested a range of machine learning models, including eXtreme Gradient Boosting (XGBoost) [21], Light Gradient Boosting Machine (LightGBM) [22], Random Forest (RF) [23], k-Nearest Neighbors (KNN) [24], and Support Vector Machine (SVM) [25]. Additionally, we incorporated deep learning models such as Multi-Layer Perceptrons (MLP) [26], Convolutional Neural Networks (CNN) [26], Recurrent Neural Networks (RNN) [27], and Transformer-based architectures [28] to explore the capacity of these frameworks for representation learning.

To ensure more stable comparisons across repeated trials, we examined several experimental variables under a controlled variable design. All experiments were repeated using three different random seeds to reduce variability, while hyperparameters for machine learning models were optimized via grid search to achieve locally optimal performance. This rigorous experimental design not only enabled fair comparisons between encoding strategies but also allowed us to identify factors associated with model performance, such as sample size, preprocessing methods, and model complexity.

By integrating a diverse set of methods, tasks, and experimental conditions, this benchmarking framework offers a comprehensive evaluation of encoding strategies for non-coding SNPs. It provides insights into how these strategies can be tailored for specific tasks, ultimately contributing to more effective genomic prediction models.

### 2.2. Quantifying Multi-Dimensional Encoding Strategies for Non-Coding SNPs

To comprehensively evaluate encoding strategies for non-coding SNPs, we examined their performance across multiple dimensions, as summarized in Table 1 and Table 2. Our analysis revealed distinct patterns in abundance, differentiation, computational cost, and interpretability, providing insights into the trade-offs inherent in these approaches and the mechanisms behind their effectiveness.

The abundance of an encoding strategy, defined as the amount of information required to describe a given mutation, varied significantly across methods. Categorical strategies such as one-hot encoding used minimal representation dimensions (4), whereas functional strategies like Enformer required significantly more (5313 dimensions). Semantic strategies, including DNABert2 (768 dimensions) and NT (1280 dimensions), struck a balance, offering moderately compact yet information-rich representations. Differentiation, defined as the change in embedding information per unit mutation length—quantifying the encoding’s ability to amplify the effect of mutations—exhibited notable variability across strategies. For instance, DNABert2 demonstrated the highest differentiation score (9.31), while Enformer exhibited a low score (0.0241) despite having the highest abundance. This highlights the diversity of encoding mechanisms, where higher abundance does not necessarily imply a stronger amplification of mutation effects as measured by differentiation.

Computational efficiency was another critical factor, particularly for large-scale genomic studies. One-hot encoding was the most computationally efficient, with a negligible time cost of 1.9978 × 10^−7^ CPU seconds per base. In contrast, Enformer was the most computationally demanding, with a normalized time cost of 8.8423 × 10^−2^ CPU seconds per base, reflecting the computational burden of generating high-dimensional functional representations. Among semantic strategies, DNABert2 achieved a favorable balance with a time cost of 1.0755 × 10^−3^, while HyenaDNA offered slightly faster embeddings at 6.8183 × 10^−5^.

Interpretability, a cornerstone of biological utility, was examined across three dimensions: positional, functional, and semantic. Positional interpretability refers to an encoding strategy’s ability to preserve explicit sequence positional information, enabling precise alignment of variant loci with genomic regulatory elements. This characteristic was present in one-hot encoding as well as in certain semantic embedding methods such as GPN and HyenaDNA (Table 2), where the encoded representations maintain a base-wise correspondence with the original DNA sequence. These strategies are particularly advantageous in tasks requiring spatial resolution of variant effects.

To further explore this property, we conducted a kernel density analysis of embeddings derived from these three positionally interpretable methods, comparing their distributions across enhancer and non-enhancer regions (Figure S2). The resulting distributions revealed that these embeddings effectively retained locational information and facilitated integrative analyses with functional genomic annotations. In particular, one-hot encoding exhibited markedly non-uniform density patterns between enhancer and non-enhancer regions. This pattern echoes the nucleotide composition biases commonly observed between functional and non-functional genomic segments [29], reinforcing its capacity to capture fine-grained, sequence-level positional variation.

Functional interpretability, defined as the incorporation of explicit genomic annotations into the representation, was uniquely achieved by Enformer, which integrates multi-modal biological context, thereby enhancing downstream functional inference. In contrast, semantic interpretability, the capacity to encode abstract and high-order biological signals, was a defining feature of semantic embedding strategies such as DNABert2, NT, and HyenaDNA. These approaches captured complex dependencies within DNA sequences, providing nuanced insights into the regulatory roles of non-coding variants beyond surface-level annotations.

These findings lead to several important conclusions. First, the relationship between abundance and differentiation is non-linear, emphasizing the importance of how information is structured within embeddings rather than the sheer dimensionality of representation. Semantic strategies like DNABert2 demonstrate this principle by achieving high differentiation with moderate abundance. Second, computational cost presents practical trade-offs, particularly for large-scale studies. While one-hot encoding remains the fastest, its limited interpretability might restrict its utility for complex tasks. Semantic strategies like DNABert2 and HyenaDNA offer practical alternatives, balancing efficiency with robust differentiation and interpretability.

Finally, no single strategy excels across all dimensions, reflecting the complementary strengths of these approaches. Functional strategies like Enformer provide unparalleled biological insights at the cost of computational efficiency, while semantic strategies capture abstract sequence relationships with less emphasis on functional annotation. This diversity underscores the potential for integrating multiple strategies to enhance both predictive performance and biological interpretability.

### 2.3. Evaluating Performance in Predicting Regulatory Directions of Non-Coding SNPs on Gene Expression

The expression quantitative trait loci (eQTL) sign prediction task focuses on classifying whether non-coding SNPs positively or negatively regulate gene expression. This task provides critical insights into the functional roles of non-coding variants in transcriptional regulation [13]. To systematically evaluate performance, we designed controlled variable experiments using eQTL data from the GTEx v8 dataset [30] for three tissues: esophagus mucosa, heart left ventricle, and nerve tibial. Five machine learning models and four deep learning models were tested, paired with six encoding strategies. Additionally, we assessed the impact of data preprocessing strategies, including chromosome-based splitting versus randomized splitting of training, validation, and testing datasets.

**Figure 2:**
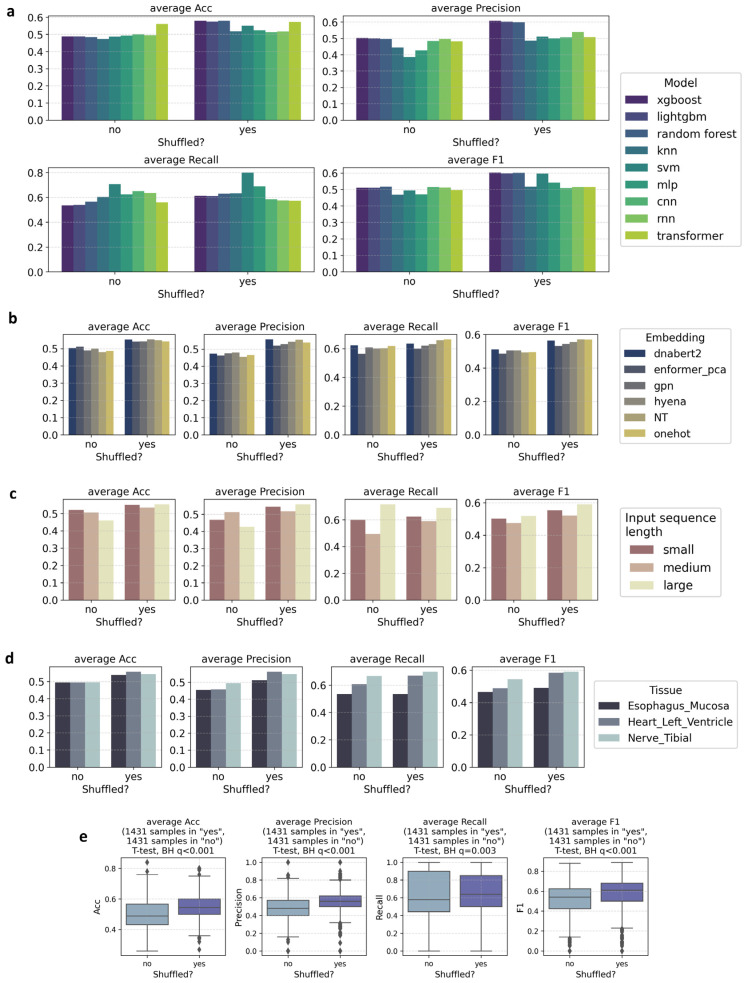
Performance analysis of eQTL sign prediction using controlled variable experiments, grouped by preprocessing strategy (“shuffled?” = “yes” indicates random splitting into training, validation, and testing sets; “shuffled?” = “no” indicates chromosome-based splitting). (**a**) Performance of classification tasks with the downstream machine learning/deep learning models as the primary variable. (**b**) Performance of classification tasks with the encoding strategy as the primary variable. (**c**) Performance of input sequence length with the encoding strategy as the primary variable. The model is divided into small (distance less than 1 kbp), medium (distance between 1 kbp and 10 kbp), and large (distance between 10 kbp and 100 kbp) according to the distance from TSS to SNP. (**d**) Performance of classification tasks with the tissue type as the primary variable. (**e**) Boxplot summarizing the impact of shuffle strategies on performance. Two-sided independent two-sample *t*-test with Benjamini–Hochberg correction results indicate that differences in shuffle strategies significantly affect the performance of this downstream task.

Machine learning models, optimized via grid search, demonstrated overall superior performance compared to deep learning models. This trend suggests that deep learning does not universally outperform traditional machine learning, particularly in datasets with limited sample sizes, such as the eQTL dataset used in this study. However, the difference in performance may also reflect the relative simplicity of the task, which machine learning models can address effectively with fewer parameters (Figure 2a and Supplementary Figure S1).

In the evaluation of different encoding strategies, we observed minimal variation in performance across embeddings. DNABert2 and NT embeddings, despite being reduced via mean pooling, achieved performance comparable to or even slightly better than one-hot encoding. This result highlights the potential of semantic embeddings in capturing meaningful sequence information, even after dimensionality reduction. Conversely, Enformer embeddings, which were reduced via principal component analysis (PCA) [31] to balance computational costs, did not outperform simpler encodings. This may be attributed to the inherent complexity of the Enformer model and the potential loss of information during dimensionality reduction (Figure 2b).

The regulatory distance between SNPs and the transcription start sites (TSS) emerged as another important factor. Although longer regulatory relationships are theoretically more challenging to model [32], our results did not show a consistent decline in performance with increasing sequence length. Instead, models trained on longer sequences (10–100 kbp) performed comparably to those with shorter inputs. This counterintuitive observation may be explained by differences in data availability; larger datasets for longer sequences likely contributed to improved training and overall performance (Figure 2c and Supplementary Figure S1).

Performance also varied across tissues, reflecting the tissue-specific nature of gene regulation and the diversity of eQTL datasets. Differences in data distribution and regulatory complexity among tissues likely influenced these results (Figure 2d).

We further examined the impact of preprocessing strategies on model performance by comparing two commonly used data partitioning methods: chromosome-based splitting and random shuffling of eQTL datasets. Statistical analysis using the two-sided independent two-sample t-test revealed that random shuffling was associated with higher performance across most models and encoding strategies (Figure 2e). However, this improvement does not necessarily imply that random shuffling is universally superior. The observed differences likely stem from the inherent structure of genomic data. Chromosome-based splitting preserves inter-chromosomal boundaries, thereby more closely simulating real-world generalization tasks where unseen chromosomal regions must be predicted. However, this approach may introduce distributional shifts between training and test sets, especially if regulatory elements or variant patterns are unevenly distributed across chromosomes. In contrast, random shuffling ensures that training and test data are drawn from similar sequence distributions, which may mitigate overfitting to chromosome-specific features and enhance apparent model performance.

These findings underscore the importance of aligning preprocessing choices with the intended use case. For rigorous performance evaluation and to avoid information leakage, chromosome-based splitting remains the more conservative and biologically realistic strategy. However, if the goal is to assess general model robustness or maximize predictive accuracy in a cross-genomic context, random shuffling may be appropriate. We recommend that future studies explicitly report their data splitting strategy and consider the trade-offs it entails in genomic modeling tasks.

### 2.4. Evaluating Performance in Predicting Impact Magnitude of Non-Coding SNPs on Gene Expression

The task of predicting the magnitude of non-coding SNPs’ impact on gene expression is a regression problem that aims to estimate the degree of gene expression change associated with specific variants. We applied the same experimental setup and data as in the previous eQTL sign prediction task, including the GTEx eQTL datasets from esophagus mucosa, heart left ventricle, and nerve tibial tissues. Similar to the classification task, machine learning models, especially those optimized through grid search, outperformed deep learning models (except for KNN, Supplementary Figure S3). Dimensionality reduction techniques, such as PCA for Enformer and mean pooling for DNA pre-trained language models, were used to manage computational costs.

The overall performance trends were consistent with those observed in the sign prediction task (Supplementary Figure S3). The impact of encoding strategies on performance remained minimal, with semantic embeddings like DNABert2 and NT showing potential despite dimensionality reduction. Additionally, the SNP-TSS distance did not significantly affect model performance, likely due to the larger data volumes available for longer sequences. As with the previous task, random shuffling of datasets significantly enhanced performance, underscoring its importance for robust training and evaluation. These results confirm the robustness of our findings from the classification task, emphasizing the effect of shuffle strategies and highlighting the potential of semantic encoding strategies.

### 2.5. Evaluating Performance in Predicting Impact Magnitude of Non-Coding SNPs on DNA Methylation Levels

We then focus on evaluating the performance of different encoding strategies in predicting the impact magnitude of non-coding SNPs on DNA methylation levels, specifically through meQTL slope prediction. For this task, we utilized the meQTL EPIC dataset [33], which includes CD4+ T cells and monocytes. As with the eQTL slope prediction, the overall performance trends follow similar patterns across different models and strategies.

Machine learning models, particularly those optimized via grid search, continued to perform robustly across most settings except for KNN (Supplementary Figure S4). Notably, the performance gap between machine learning and deep learning models was smaller in this task, potentially reflecting the stabilizing effect of a larger dataset. This suggests that while machine learning models offer consistent performance across varying conditions, deep learning architectures may hold greater potential under settings with higher data volume and increased regulatory complexity.

In terms of encoding strategies, Enformer embeddings—despite dimensionality reduction via PCA—achieved the highest Pearson correlation in some scenarios, suggesting that biologically annotated representations may retain advantageous functional signals for methylation prediction. At the same time, semantic embeddings such as DNABert2 and NT remained competitive, even after mean pooling, highlighting their robustness and adaptability across downstream tasks. The variation in model performance across cell types further reflects the diversity of SNP–CpG interactions and the cell-type-specific regulatory mechanisms embedded in the meQTL landscape.

### 2.6. Univariate Analysis: Investigating the Relationship Between Model Complexity, Tissue Type, and Performance

**Figure 3:**
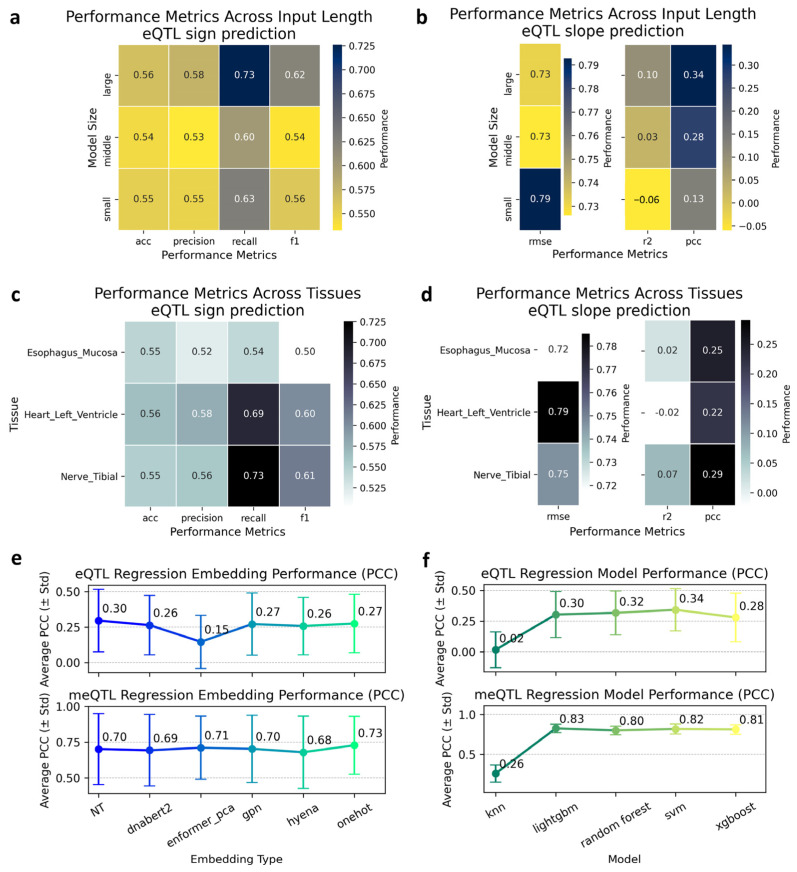
Univariate analysis of QTL prediction tasks. (**a**–**d**) Investigation of the model performance with input sequence length and tissue type as observed explicit variables and corresponding sample size as the implicit variable. (**e**,**f**) Examination of the relationship between embeddings, downstream task modeling strategies (machine learning), and model performance on slope prediction tasks.

In QTL prediction tasks, longer genomic regions are often expected to introduce more complex regulatory relationships, which may increase the difficulty of prediction. In our eQTL sign prediction task, however, we observed that the large input setting showed higher performance than the medium input setting in several comparisons (Figure 3a,b). This observation should be interpreted cautiously, because input length was not independent of dataset composition in the present benchmark. In particular, the large input setting contained more samples than the medium input setting, which may have improved model fitting and contributed to the apparent performance advantage (Supplementary Figure S1c). Thus, the observed trend is correlational and does not establish a causal effect of input length or model scale.

To further explore this, we examined whether the performance differences could be attributed to sample size or the inherent differences between tissue types. Specifically, we compared the data volumes used for training across various tissues (Supplementary Figure S1a) and found that the heart left ventricle tissue, with the smallest dataset, did not necessarily exhibit the worst model performance (Figure 3c,d). This suggests that while sample size may have an impact, tissue type itself could also be influencing model performance. The variation in regulatory mechanisms across tissues likely leads to differences in fitting difficulty and generalization, highlighting the complex interplay between sample size, tissue type, and prediction performance.

### 2.7. Cross-QTL Task Analysis: Exploring the Effectiveness of Encoding Strategies and Models

To further understand the impact of encoding strategies and model choices on QTL prediction tasks, we conducted cross-QTL task analysis by examining the effectiveness of different embeddings in slope prediction (Figure 3e,f). Here, cross-QTL task analysis refers to comparing the overall behavior of encoding strategies and model types across different QTL prediction tasks, rather than integrating eQTL and meQTL variants at matched genomic coordinates.

In this experiment, we compared the performance of various encoding strategies, across two regression tasks. Interestingly, the performance differences between encoding strategies in the meQTL slope prediction task were relatively minor. Given the large sample size of the meQTL dataset, it is likely that all embedding strategies were sufficiently powerful to capture the underlying regulatory patterns, resulting in a comparable model performance (Figures S1 and S5).

In contrast, when applied to the eQTL tasks, performance discrepancies were more evident, particularly when Enformer was reduced via PCA to fit the task’s requirements. This suggests that dimensionality reduction, while necessary to handle the high computational cost of Enformer, may hinder its effectiveness in tasks with smaller sample sizes, such as eQTL. In terms of modeling approaches, tree-based algorithms (XGBoost, LightGBM, and Random Forest) performed consistently well across the tasks. SVM showed similar performance to the tree models, but the KNN algorithm underperformed. KNN’s poor performance in QTL prediction tasks can be attributed to its reliance on local similarity and inability to capture the complex, non-linear relationships and feature interactions present in high-dimensional genomic data.

### 2.8. Guided Analysis for Practical Use: Case Study on meQTL Prediction

**Figure 4:**
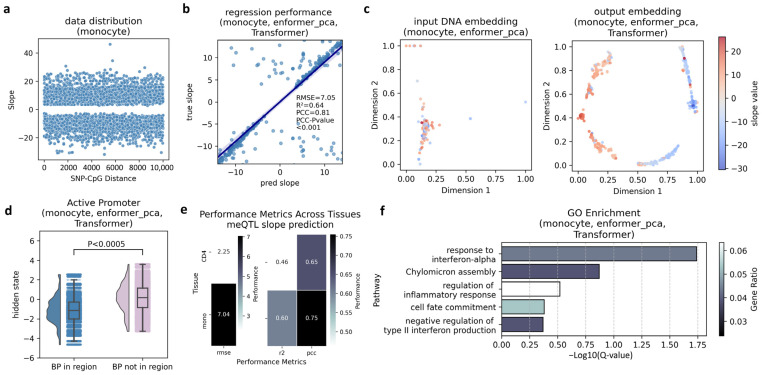
Case study on meQTL prediction as a guided analysis for practical use. (**a**) Visualization of the data distribution for monocyte meQTL to provide an intuitive overview of the dataset. (**b**) Performance evaluation of a regression task on the test set using PCA-reduced Enformer embeddings as input and a Transformer framework for representation learning. (**c**) Visualization of embeddings before and after model input using t-SNE dimensionality reduction, showcasing the model’s representation learning capability. (**d**) Analysis of the model’s interpretability by annotating hidden states corresponding to bases located in active promoter regions. (**e**) Evaluation of model performance across different cell types, with data volume as the implicit variable. (**f**) GO enrichment analysis of genes associated with SNPs predicted to have relatively large methylation effects using an exploratory cutoff of |slope| > 0.2.

In the case study, we aimed to offer a guided analysis for practical use in meQTL prediction, providing a comprehensive framework for how one might approach QTL-related tasks. This study is not only a showcase of the potential of our framework but also serves as a reference for others looking to implement QTL prediction with genomic data. We began by visualizing the data distribution for monocyte meQTL, which provided an intuitive overview of the dataset, helping contextualize the performance of subsequent analysis (Figure 4a). For the performance evaluation, we employed a regression task using PCA-reduced Enformer embeddings as input and a Transformer framework for representation learning. This allowed us to assess the model’s predictive power on the test set (Figure 4b).

Further investigation into the model’s behavior was conducted by visualizing the embeddings before and after model input with t-SNE dimensionality reduction, revealing how well the model learned meaningful representations of the data (Figure 4c). We also performed an interpretability analysis by annotating hidden states corresponding to bases located in active promoter regions, highlighting the model’s ability to link genomic features with regulatory functions (Figure 4d). Additionally, we evaluated model performance across different cell types, observing how varying data volumes influenced the results (Figure 4e).

To further connect model predictions with downstream biological interpretation, we performed Gene Ontology (GO) enrichment analysis [34] on genes associated with SNPs predicted to have relatively large methylation effects. In this case study, we used an absolute predicted slope greater than 0.2 as a practical effect-size filter to focus the analysis on variants with stronger predicted effects. The enriched terms provided functional context for these candidate variants and suggested biological processes that may be worth further investigation. This analysis illustrates how the benchmarked prediction pipeline can be extended from model evaluation to exploratory functional annotation.

This case study illustrates how the benchmark can support practical methodological decisions in meQTL prediction. First, visualizing the target distribution helps assess whether the regression task is dominated by near-zero effects or contains sufficient high-effect samples for downstream modeling. Second, comparing model performance across cell types provides a way to evaluate whether a selected encoding-model combination is stable under different data volumes and regulatory contexts. Third, representation visualization and promoter-region annotation offer exploratory checks on whether the learned features retain biologically relevant sequence or regulatory context. Finally, GO enrichment analysis of genes associated with SNPs with relatively large predicted methylation effects provides a hypothesis-generating functional annotation step.

## 3. Discussion

In this study, we explored and quantified the performance of various encoding strategies and models in predicting the regulatory effects of non-coding SNPs. By evaluating multiple encoding strategies, including categorical, semantic, and functional embeddings, across several downstream tasks, we identified the effectiveness and potential trade-offs associated with each strategy in different genomic contexts. Focusing on tasks such as eQTL and meQTL prediction, we employed a controlled variable approach to ensure the robustness and reliability of our findings. Our comparison of machine learning and deep learning models further highlighted the nuanced interplay between model choice and data characteristics, such as sample size and machine/deep learning models. Moreover, the analysis of preprocessing strategies emphasized the critical role of dataset splitting in ensuring accurate model evaluation. This work provides insights for advancing the analysis of non-coding SNPs, ultimately contributing to a deeper understanding of their biological roles.

Several practical considerations emerge from this benchmark. First, data splitting strategy should be explicitly reported because random shuffling and chromosome-based splitting can lead to different performance estimates. Second, chromosome-based splitting may provide a more conservative evaluation setting for assessing generalization to unseen genomic regions. Third, representation complexity should be balanced against computational cost, as higher-dimensional or functionally annotated embeddings do not necessarily lead to better downstream performance after dimensionality reduction. Fourth, in limited-data QTL settings, traditional machine learning models may remain competitive with deep learning models, and model complexity should be selected according to dataset size and task characteristics.

The case study on meQTL prediction demonstrated the practical utility of the methods developed in this work, providing insights for researchers in the field. Our guided analysis also exemplified how the interpretability of models can be enhanced through visualizations such as t-SNE and by annotating hidden states, offering a more contextual view of predicted SNP effects on methylation. This comprehensive approach not only contributes to advancing meQTL prediction but also sets the stage for future work aimed at refining predictive models, integrating additional genomic information, and scaling these methods to broader datasets.

It should be noted that the relatively higher performance of machine learning models in our experiments does not imply an intrinsic advantage over deep learning frameworks. Instead, this difference likely reflects the limited data size and the absence of large-scale architecture or hyperparameter searches in the present benchmark. Under more extensive optimization or larger datasets, deep learning models may demonstrate superior representation capacity. The GO enrichment analysis in the case study should be interpreted cautiously because the |slope| > 0.2 threshold was used as an exploratory cutoff for candidate selection. The resulting enriched terms provide functional hypotheses for future validation rather than direct evidence of disease mechanisms.

The field of DNA and genomic foundation models is advancing rapidly, and several recently developed models were not included in the present benchmark. For example, Evo [11] extends genome-scale language modeling across diverse organisms and supports long-context sequence modeling at single-nucleotide resolution. AlphaGenome [35] provides a unified sequence-to-function framework for predicting diverse regulatory genomic tracks and variant effects from long DNA sequences. CrossDNA [36] further highlights the importance of explicitly modeling double-strand and reverse-complement relationships in DNA representation learning. These models may offer additional advantages for long-range regulatory modeling, variant-effect prediction, and strand-aware sequence representation. The present study focused on representative encoding strategies across categorical, semantic, and functional paradigms, rather than an exhaustive comparison of all available DNA foundation models. Future extensions of this benchmark will incorporate these emerging models to evaluate whether their architectural advances translate into improved performance in QTL-related prediction tasks.

While this benchmark provides valuable insights into the comparative performance of various encoding strategies and models, several limitations remain. First, while the present study focuses on three GTEx tissues and two meQTL cell types selected for data quality and consistency, we acknowledge that these contexts do not encompass the full biological diversity of human regulatory landscapes. Future work will extend this framework to additional tissues such as brain and liver, as well as to disease-relevant cellular systems like cancer cell lines, to comprehensively evaluate the generalizability of encoding strategies across biological contexts. Additionally, while we have assessed a variety of encoding methods and models, there may exist other, more optimal combinations or novel encoding approaches that were not included in this study, warranting further exploration. Future work could also focus on improving the performance of models on small sample sizes and enhancing their generalization capabilities.

Another limitation is that comparisons across input-length settings may be partially confounded by differences in sample size and dataset composition. Therefore, the observed performance trends should be interpreted as associations within the current benchmark rather than causal evidence for the superiority of a particular input length. Future work with sample-size-matched or down-sampled experimental designs will be needed to more rigorously assess the independent effect of input sequence length.

Ultimately, this benchmark establishes a foundational framework for future studies on non-coding SNPs, offering insights into the design of predictive pipelines and the selection of appropriate models for specific genomic tasks.

## 4. Materials and Methods

### 4.1. Datasets

For eQTL data, we downloaded eQTL for three tissues: esophagus mucosa, heart left ventricle, and nerve tibial from the GTEx v8 dataset [30]. These datasets were used to evaluate the regulatory effects of non-coding SNPs on gene expression across diverse genomic contexts. For meQTL data, we utilized the meQTL EPIC dataset, which included meQTL data for two cell types: CD4+ T cells and monocytes. The distributions of these datasets are visualized in Supplementary Figure S1.

In this study, eQTL datasets were annotated and DNA sequences were retrieved based on the GRCh38/hg38 reference genome, while meQTL datasets were processed using the GRCh37/hg19 reference genome. All sequence extraction, annotation, encoding, and downstream modeling were conducted within each dataset’s own genome-build framework. We did not perform locus-level integration, coordinate matching, or direct variant-level comparison between eQTL and meQTL datasets. Therefore, cross-build coordinate conversion was not required for the analyses presented in this benchmark. Genomic sequences and their functional annotations were obtained from the UCSC Genome Browser, ensuring consistency in annotation and feature extraction. All analyses were conducted within each dataset’s coordinate framework without cross-build alignment, ensuring internal consistency.

For datasets without random shuffling, we used a chromosome-based splitting strategy during model training. Specifically, the training set consisted of data from chromosomes 1–9 and 14–22, the validation set was derived from chromosomes 12–13, and the test set was sourced from chromosomes 10–11. This chromosome-based partition followed the held-out chromosome evaluation strategy used in CpGenie-related work [12].

### 4.2. Encoding Strategies

In this study, all encoding strategies require DNA sequence sampling, with the sampling standard differing between tasks. For eQTL prediction tasks, the sequence is sampled with the TSS at the center, ensuring the mutation is within the sampling range. For meQTL prediction tasks, the sequence is sampled with the CpG site at the center, ensuring the mutation is included in the sampling range. Specifically, for eQTL prediction, sequence lengths of 2001 bp, 20,001 bp, and 200,001 bp are used, while for meQTL tasks, a sampling length of 20,001 bp is applied.

- OneHot. The DNA sequence is represented by a one-hot encoded vector with four possible values corresponding to the four bases (A, T, C, G). If a base is ‘N’ (unknown), it is encoded as four zeros.
- DNABert2 [14]. DNABert2 is a pre-trained DNA language model, which was implemented using the official API (https://github.com/MAGICS-LAB/DNABERT_2, accessed on 24 December 2024) in this study. For DNABert2, we designed a position-wise average cutting and pooling strategy, aiming to maximize mutation representation. The processing steps are as follows: given a DNA sequence centered on either the TSS (for eQTL) or CpG site (for meQTL), the two outer segments are cut into lengths of 250 bp, while the center segment (501 bp) is centered around the TSS or CpG site. The remaining parts of the DNA sequence between the two outer segments and the central segment are evenly divided into 500 bp fragments. For each fragment, an embedding is generated, and embeddings are concatenated along the embedding dimension. Finally, the concatenated embeddings are averaged over the DNA sequence dimension to produce a one-dimensional vector of the same dimension as the language model embedding.
- GPN [15]. GPN is a pre-trained DNA language model, implemented using the official API (https://github.com/songlab-cal/gpn, accessed on 24 December 2024) in this study. The same position-wise average cutting and pooling strategy applied to DNABert2 is also used here.
- HyenaDNA [16]. HyenaDNA is another pre-trained DNA language model, implemented via the official API (https://github.com/HazyResearch/hyena-dna, accessed on 24 December 2024) in this study. Like DNABert2, it applies the position-wise average cutting and pooling strategy.
- NT [17]. NT is a pre-trained DNA language model, implemented using the official API (https://github.com/instadeepai/nucleotide-transformer, accessed on 24 December 2024) in this study. It also utilizes the position-wise average cutting and pooling strategy, similar to DNABert2.
- Enformer [13]. Enformer is a model designed for gene expression prediction from DNA sequences. In this study, we extracted DNA sequences of length 196,608 bp centered around the TSS or CpG sites to match Enformer’s input requirements. For both pre-mutated and post-mutated sequences, we obtained output features with a dimension of 5313. When calculating quantitative metrics, we directly utilized these output features. For Enformer representations, we applied PCA to reduce the high-dimensional output features before downstream modeling. This step was introduced to reduce computational cost and to make Enformer representations more tractable for comparison with other encoding strategies in the benchmark. We retained the top 10 principal components as a compact representation for downstream models. Because this compression may discard information from the original Enformer outputs, the Enformer-PCA results should be interpreted as performance under a computationally reduced setting rather than as the full predictive capacity of the original Enformer representation.

For Enformer, PCA was used to extract the major variance components from its high-dimensional annotation-based outputs, thereby reducing computational cost while retaining biologically meaningful information. For pre-trained DNA language models such as DNABert2, NT, and HyenaDNA, mean pooling was applied to aggregate position-wise embeddings into a single representative vector, which preserves contextual semantics and ensures dimensional consistency across models for downstream tasks.

### 4.3. Feature Profile of Encoding Strategies

To comprehensively evaluate the strengths and limitations of different encoding strategies, we defined a set of feature profiles to quantify their capabilities in representing non-coding SNPs.

- Abundance. Abundance measures the amount of information required to describe a mutation. Specifically, it refers to the dimensionality of the embedding per base, indicating how detailed the representation is for each SNP. The final value is normalized by the sequence length L, measured as the length of the DNA sequence centered on the mutation:
  $$Y_{abundance}=\frac{\prod_{i=1}^{n}d_{i}}{L}$$
  where $d_{i}$ is the dimensionality of the i-th embedding axis. For example, a 501 bp sequence encoded by one-hot contains four base channels at each nucleotide position, so the normalized abundance is reported as 4.0000. GPN generates a position-wise representation with 512 features for each base, so its abundance is reported as 512.0000. In contrast, for DNABert2, NT, and Enformer, the reported abundance values may not be identical to their raw hidden dimensions or output-track numbers, because their representations are affected by tokenization, sequence segmentation, pooling, or binned functional outputs. Thus, the abundance values in Table 1 should be interpreted as normalized representation density rather than raw embedding dimensionality.
- Differentiation. Differentiation quantifies the change in embedding information per unit length of DNA before and after mutation, effectively capturing the ability of an encoding strategy to amplify mutation effects. This difference is computed for each dimension of the embedding vector, $d_{i}$, and averaged over the entire sequence length L centered around the mutation. The formula can be expressed as:
  $$X_{before}^{\prime }=\frac{X_{before}-X_{min(before)}}{X_{max(before)}-X_{min(before)}}$$
  $$X_{after}^{\prime }=\frac{X_{after}-X_{min(after)}}{X_{max(after)}-X_{min(after)}}$$
  $$X^{\prime }=X_{after}^{\prime }-X_{before}^{\prime }$$
  $$Y_{differentiation}=\frac{\sum_{d_{1}=1}^{m_{1}}\sum_{d_{2}=1}^{m_{2}}\ldots \sum_{d_{n}=1}^{m_{n}}\left|X_{d_{1}d_{2}\ldots d_{n}}^{\prime }\right|}{L}$$
  where $X^{\prime }$ represents the normalized difference in the i-th dimension of the embedding vector before and after mutation.
- Time cost. Time cost represents the computational expense required to generate embeddings for a unit length of DNA, both before and after mutation. This metric reflects the efficiency of the encoding strategy, which is particularly critical for large-scale genomic datasets:
  $$Y_{time\;cost}=\frac{T1+T2}{L}$$
  where T1 and T2 represent the computational time required to generate the embedding vectors for the unmutated and mutated DNA sequences, respectively.
- Interpretability. Interpretability assesses whether the encoding preserves key biological information, such as the relative positions of bases or functional annotations of the DNA sequence. This dimension is crucial for understanding the biological implications of predictions made by models using these encodings.
  (1) Positional Interpretability: the clarity with which the embedding can be directly mapped to individual nucleotides in the DNA sequence. This means the position of each nucleotide in the original sequence can be unambiguously represented in the embedding.
  (2) Functional Interpretability: whether the embedding captures biological features that can be derived from experimental techniques such as sequencing. This includes elements like transcription factor binding sites, enhancers, or other functional genomic elements that are detectable through high-throughput sequencing methods.
  (3) Semantic Interpretability: refers to the extent to which embeddings learned from large-scale DNA sequence pretraining capture contextual and high-order sequence patterns that may support downstream prediction. Unlike functional interpretability, semantic interpretability does not indicate a direct correspondence to experimentally defined regulatory annotations but reflects whether the learned representations provide biologically relevant sequence context for modeling non-coding SNP effects.

In this work, we do not investigate the biological properties that might be encoded in embeddings derived solely from pre-trained DNA sequence models, as these properties are currently not directly modelable in this context. Conceptually, abundance describes how much information an encoding strategy can represent for each nucleotide, reflecting its overall richness of representation. In contrast, differentiation indicates how sensitively the encoding responds to a single-base mutation, capturing the magnitude of change in the embedding caused by that mutation. Together, these two metrics characterize both the information capacity and the mutation responsiveness of an encoding strategy.

### 4.4. Machine Learning and Deep Learning Models

For machine learning models, we selected eXtreme Gradient Boosting (XGBoost) [37], Light Gradient Boosting Machine (LightGBM) [22], Random Forest (RF) [23], k-Nearest Neighbors (KNN) [24], and Support Vector Machine (SVM) [25]. To optimize these models, we utilized the GridSearchCV function from the Scikit-learn Python library (version: 1.1), which systematically searches for the best combination of hyperparameters to achieve locally optimal solutions.

For deep learning models, we explored Multi-Layer Perceptrons (MLP) [26], Convolutional Neural Networks (CNN) [26], Recurrent Neural Networks (RNN) [27], and Transformer-based architectures [28]. These frameworks were chosen to evaluate their capacity for representation learning and their ability to model complex relationships within the genomic data. For the MLP, CNN, RNN, and Transformer baselines, the input feature was constructed by flattening and concatenating the representations of the reference and alternative sequences. For example, for GPN-based eQTL and meQTL models, the concatenated input dimension was 1024, corresponding to the 512-dimensional GPN embedding of the reference sequence and the 512-dimensional GPN embedding of the alternative sequence. For the one-hot meQTL models, the concatenated input dimension was 160,008, corresponding to 20,001 bp, four one-hot channels, and two alleles. For models using DNABERT, Nucleotide Transformer, HyenaDNA, GPN, or Enformer-PCA representations, the input dimension was determined by the corresponding flattened before- and after-mutation embeddings.

The MLP baselines were implemented using scikit-learn, with three hidden layers of 1024, 512, and 128 units. The CNN model contained two one-dimensional convolutional blocks followed by a task-specific output layer, while the RNN model used a SimpleRNN layer followed by a task-specific output layer. The Transformer baselines were implemented with a 6-layer Transformer encoder, 8 attention heads, mean pooling, and a classification or regression head. Classification models used sigmoid or softmax-based outputs with cross-entropy loss, whereas regression models used linear outputs with mean squared error loss. MLP models were trained with a maximum of 1000 iterations and early stopping. CNN and RNN models were trained with Adam, validation-loss-based early stopping, and a maximum of 1000 epochs. Transformer models were trained with Adam for 100 epochs. The held-out test set was used only for final evaluation.

To ensure the robustness of our findings, all experiments were conducted with three different random seeds, reducing variability and enhancing the reliability of our conclusions. This systematic approach allowed for a comprehensive comparison between machine learning and deep learning models in the context of QTL prediction tasks.

### 4.5. Computational Environment

All data preprocessing and machine learning analyses were performed on a CPU computing cluster equipped with dual Intel^®^ Xeon^®^ Platinum 8358 processors (32 cores each). To ensure fair and consistent benchmarking, all embeddings were generated on CPU resources. Deep learning experiments were carried out on a workstation with an NVIDIA GeForce RTX 3090 GPU. No distributed or parallel computing was employed.

### 4.6. Performance Evaluation

For the classification task, we employed Accuracy (ACC), Precision, Recall, and F1-score to assess model performance. For the regression task, we utilized Root Mean Square Error (RMSE) and Pearson Correlation Coefficient (PCC) as key metrics.

## Figures and Tables

**Table 1:** Feature profile of encoding strategies.

Encoding Group	Encoding Strategy	Normalized Abundance (Representation Elements per Base)	Differentiation	Normalized Time Cost (CPU Seconds per Base)
Categorical	One-Hot	4.0000	0.0040	1.9978 × 10^−7^
Semantic	DNABert2 [14]	469.5377	9.3144	1.0755 × 10^−3^
Semantic	GPN [15]	512.0000	1.3086	5.8716 × 10^−3^
Semantic	HyenaDNA [16]	128.5110	0.5234	6.8183 × 10^−5^
Semantic	NT [17]	1277.4451	4.6028	1.3727 × 10^−2^
Functional	Enformer [13]	9501.8922	0.0241	8.8423 × 10^−2^

**Table 2:** Interpretability analysis of encoding strategies.

Encoding Group	Encoding Strategy	Positional	Functional	Semantic
Categorical	One-Hot	Yes	No	No
Semantic	DNABert2 [14]	No	No	Yes
Semantic	GPN [15]	Yes	No	Yes
Semantic	HyenaDNA [16]	Yes	No	Yes
Semantic	NT [17]	No	No	Yes
Functional	Enformer [13]	No	Yes	No

## Data Availability

The tissue-specific eQTL data were collected from the GTEx v8 portal [30] (https://www.gtexportal.org/home/datasets, accessed on 14 May 2024). The meQTL EPIC dataset [33] was downloaded from the meQTL EPIC Database website (https://epicmeqtl.kcl.ac.uk/, accessed on 14 May 2024). The GRCh37/hg19 genome, GRCh38/hg38 genome, and functional annotation were obtained from the UCSC Genome Browser (https://genome.ucsc.edu/, accessed on 14 May 2024). The CpG annotation of Infinium MethylationEPIC v1.0 B5 manifest file was downloaded from https://support.illumina.com/downloads/infinium-methylationepic-v1-0-product-files.html (accessed on 14 May 2024). The source codes of the preprocessing, modeling, and validation processes are freely available on GitHub (https://github.com/Liuzhe30/DNAMutBenchMark, accessed on 14 May 2024).

## Supplementary Materials

The following supporting information can be downloaded at: https://www.mdpi.com/article/10.3390/ijms27156657/s1.
