# Supplementary material for: Systematic Benchmarking of DNA Sequence Encoding Strategies for Predicting Regulatory Effects of Non-Coding SNPs
Source: Int J Mol Sci. 2026 Jul 25;27(15):6657. doi: 10.3390/ijms27156657 (PMC13465526; doi:10.3390/ijms27156657)

Supplementary Information

Figures

Figure S1.

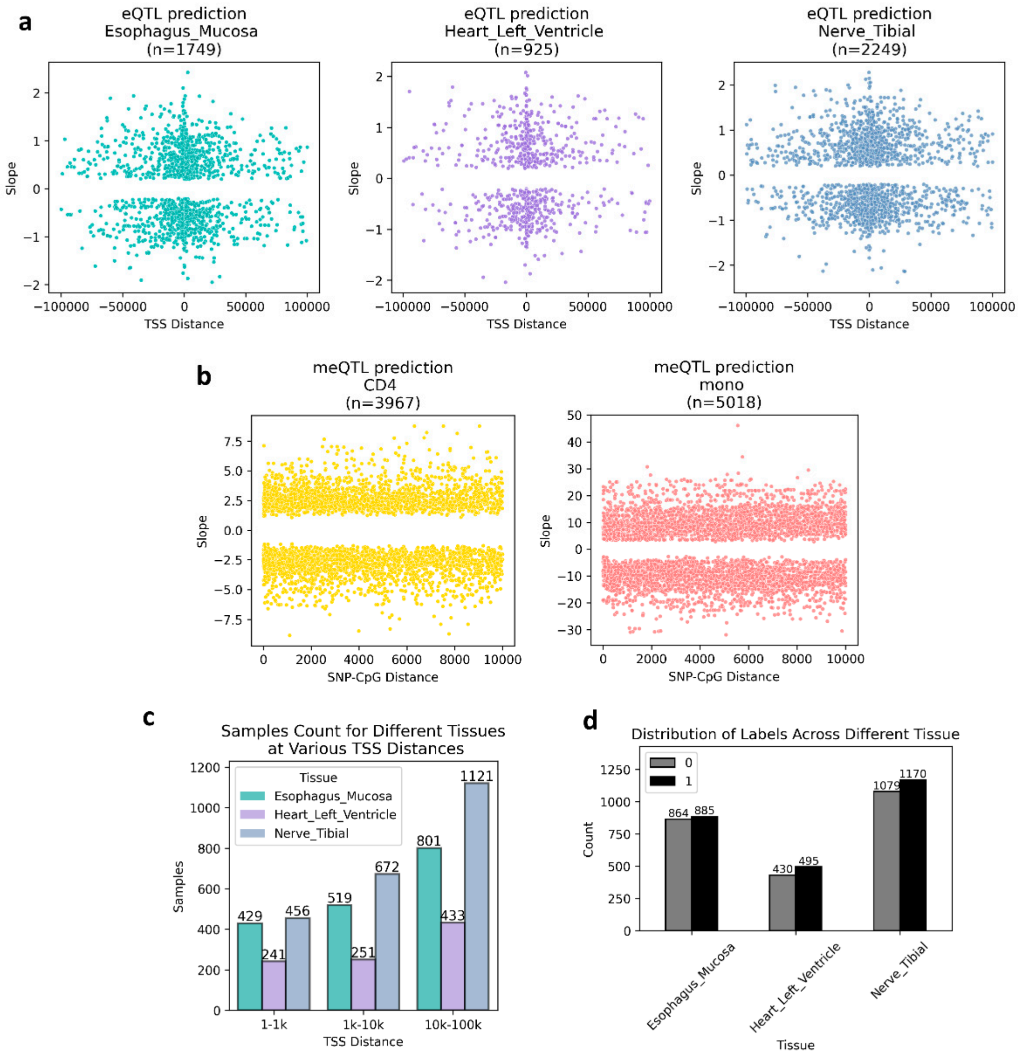

Figure S1. The data distribution of the benchmark datasets in pre-processing progress.

(a) Data distribution of the eQTL datasets.

(b) Data distribution of the meQTL datasets.

(c) Sample count for different tissues at various TSS distance.

(d) Distribution of labels across different tissue.

Figure S2.

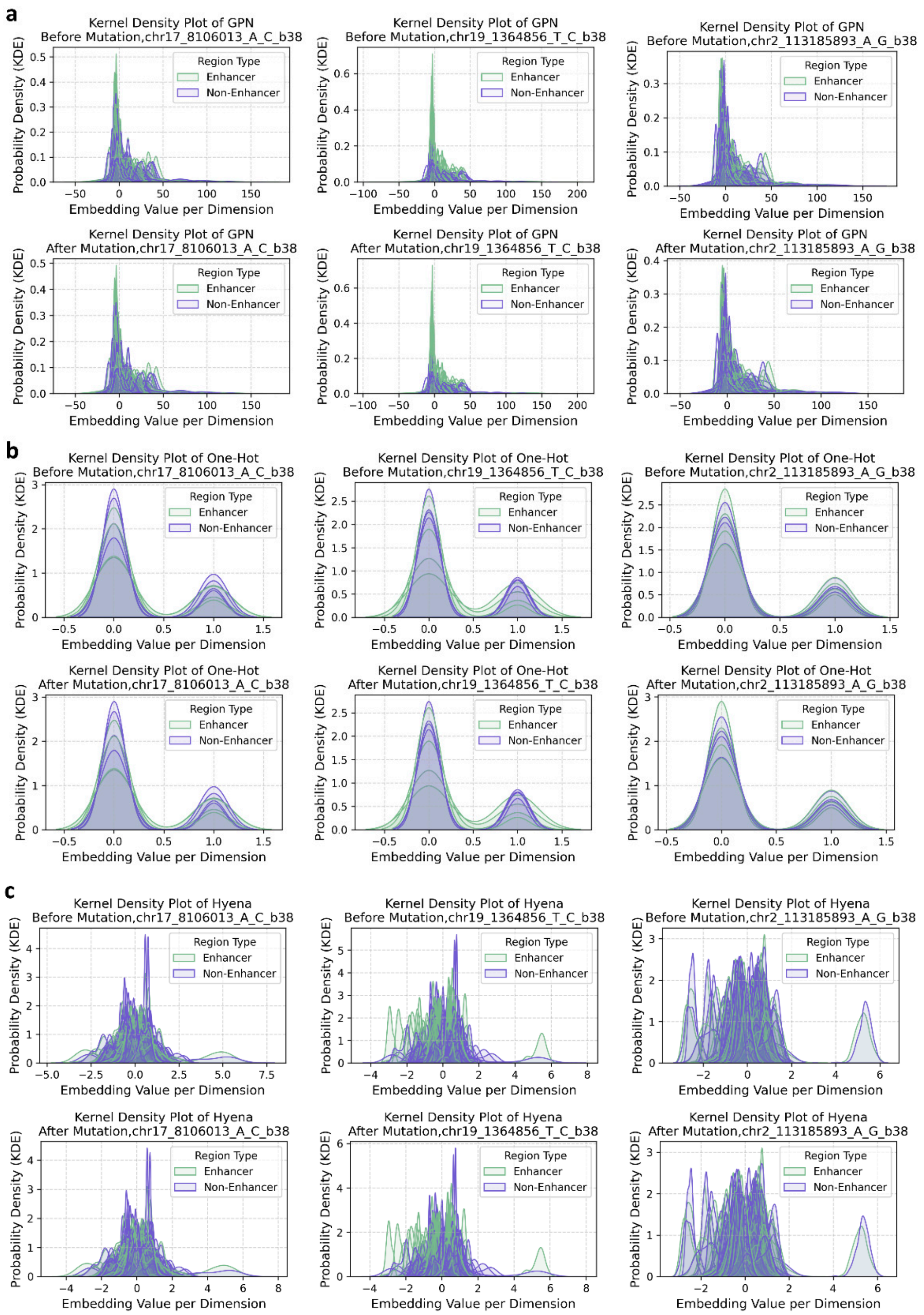

Figure S2. Kernel density plots of three positionally interpretable coding strategies in enhancer and non-enhancer regions. (a) Kernel density plot of GPN embeddings for enhancer and non-enhancer regions, illustrated using three example SNPs. (b) Kernel density plot of One-Hot embeddings for enhancer and non-enhancer regions, illustrated using three example SNPs. (c) Kernel density plot of Hyena embeddings for enhancer and non-enhancer regions, illustrated using three example SNPs.

Figure S3.

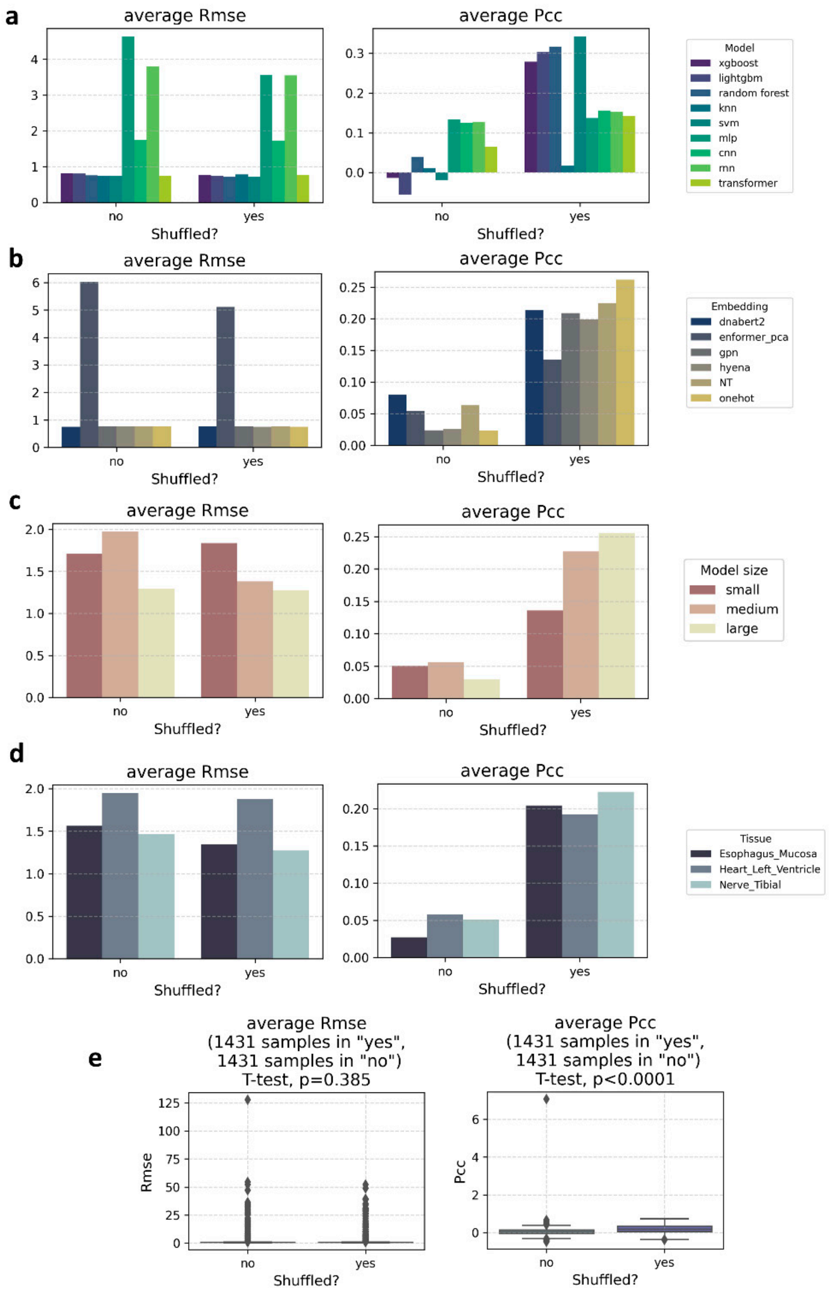

Figure S3. Performance analysis of eQTL slope prediction using controlled variable experiments, grouped by preprocessing strategy ("shuffled?" = "yes" indicates random splitting into training, validation, and testing sets; "shuffled?" = "no" indicates chromosome-based splitting). (a) Performance of machine learning/deep learning models as the primary variable. (b) Performance of the encoding strategy as the primary variable. (c) Performance of input sequence length with the encoding strategy as the primary variable. (d) Performance of the tissue type as the primary variable. (e) Boxplot summarizing the impact of shuffle strategies on performance.

Figure S4.

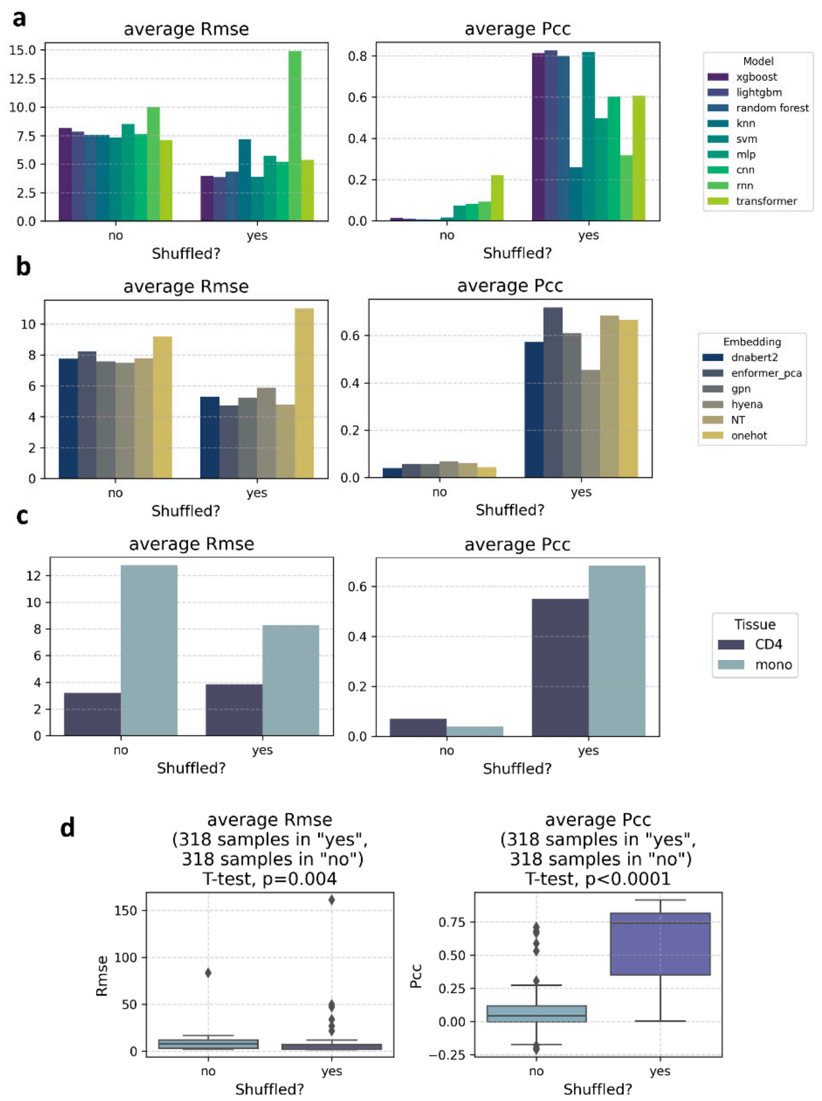

Figure S4. Performance analysis of meQTL slope prediction using controlled variable experiments, grouped by preprocessing strategy ("shuffled?" = "yes" indicates random splitting into training, validation, and testing sets; "shuffled?" = "no" indicates chromosome-based splitting). (a) Performance of machine learning/deep learning models as the primary variable. (b) Performance of the encoding strategy as the primary variable. (c) Performance of input sequence length with the encoding strategy as the primary variable. (d) Performance of the tissue type as the primary variable. (e) Boxplot summarizing the impact of shuffle strategies on performance.

Figure S5. Investigation of the relationship between sample size and model performance, with model size and cell type as observed explicit variables and corresponding sample size as the implicit variable.

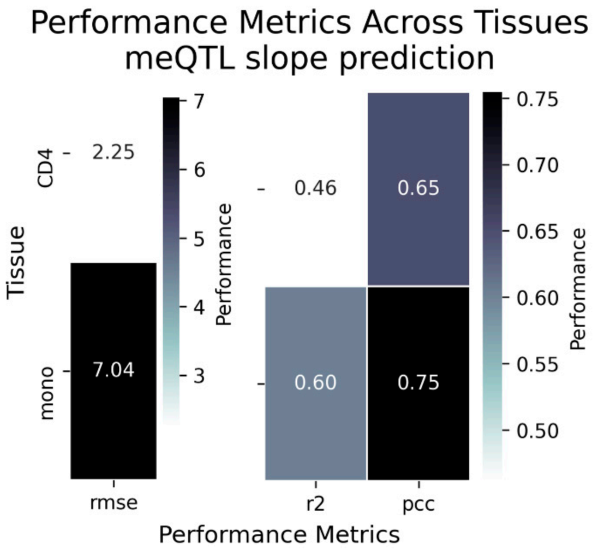

Supplement: Supplementary file 1 [file ijms-27-06657-s001.zip › ijms-4361287-supplementary.pdf]
